# Supplementary material for: The Impact of the Inoculation of Different Pied de Cuve on the Chemical and Organoleptic Profiles of Wines
Source: Microorganisms. 2024 Aug 13;12(8):1655. doi: 10.3390/microorganisms12081655 (PMC11356560; doi:10.3390/microorganisms12081655)
Supplement: Supplementary file 1 [file microorganisms-12-01655-s001.zip › microorganisms-3122700-supplementary.pdf]

Table S1. Area Under Curve (AUC) of different inoculation modalities to conduct the alcoholic fermentation (AF). Different superscript letters indicate that the values are significantly different within the same harvest (p-value < 0.05).

Harvest	AF modality	AUC
M2	P-M2-26	9206.41 ± 3.91 ^{a}
	P-M2-18	9193.40 ± 4.46 ^{ab}
	P-M2-26SE	9184.90 ± 7.39 ^{b}
	P-M2-18SE	9198.70 ± 3.52 ^{ab}
	C-M2	9203.19 ± 13.98 ^{ab}
S3	P-S3-26SE	15212.62 ± 5.69 ^{a}
	P-S3-18SE	15223.62 ± 19.73 ^{b}
	C-S3	15254.75 ± 4.16 ^{b}
M3	P-M3-18SE	18401.62 ± 9.30 ^{a}
	C-M3	18570.98 ± 9.67 ^{b}
	SF-M3	18725.48 ± 5.16 ^{c}
